# A Case Report of an Open Pan-Talar Dislocation

**DOI:** 10.7759/cureus.9274

**Published:** 2020-07-19

**Authors:** Ahmed Genena, Amr Abouelela

**Affiliations:** 1 Trauma and Orthopaedics, Faculty of Medicine, Helwan University, Alsikka Al Hadid Algharbeya, Helwan, EGY; 2 Trauma and Orthopaedics, University Hospitals of Derby and Burton, Burton, GBR

**Keywords:** open injury, talar dislocation, avascular necrosis, foot injury

## Abstract

Total talar extrusion is a rare injury that most commonly occurs secondary to high-energy trauma. There are few reported cases of open dislocations in literature and still, there is no consensus regarding the appropriate treatment of the extruded talus. In this case report, we present a 12-month follow-up of a patient with an open talar dislocation with extrusion treated with immediate surgical debridement, reduction and temporary fixation with one Steinmann pin. No infection was reported, although the patient developed avascular necrosis.

## Introduction

Pan-talar dislocation is a rare injury that accounts for nearly 0.06% of all dislocations and only 2% of all talar injuries [1,2]. It usually results from high-energy trauma such as motor vehicle accidents or falls from a height. Most commonly, these injuries are open and are associated with a high rate of complications including avascular necrosis, infection, post-traumatic arthritis and the need for secondary procedures. Historically, primary talectomy and tibiocalcaneal arthrodesis were recommended to diminish the rate of these complications; however, recent literature advocates surgical debridement and re-implantation with stabilization [3-8].

## Case presentation

**Figure 1:**
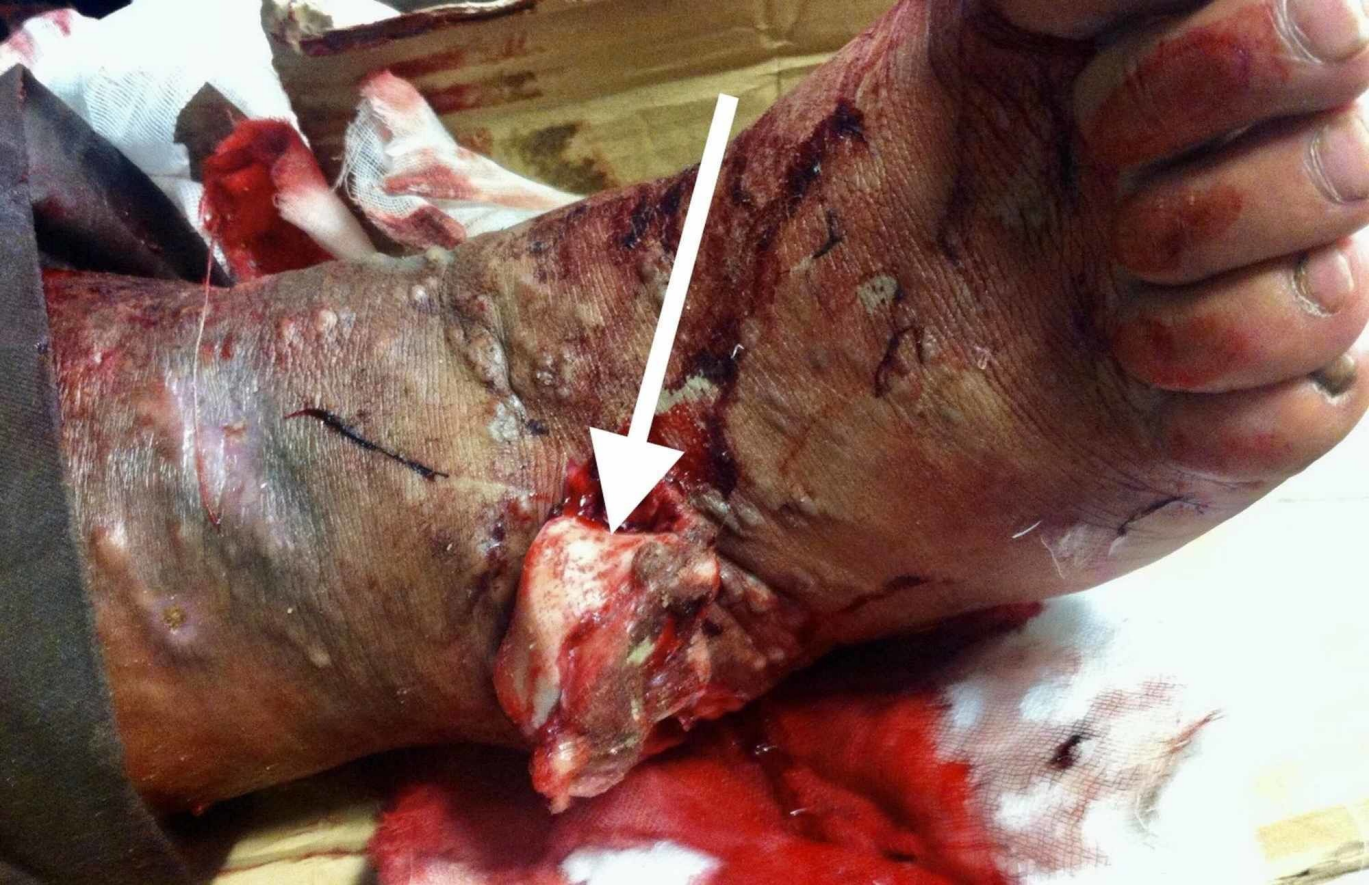
Clinical photograph of the right ankle demonstrating a total extrusion of the talus bone through an anterolateral open wound (arrow)

In December 2018, a 46-year-old male presented to the ED two hours after sustaining an open injury to his right ankle in a motor vehicle accident. After full advanced trauma life support protocol, an open right ankle fracture was the isolated injury. On further physical examination, his right talus was extruded through a 10-cm anterolateral wound (Figure 1). Both dorsalis pedis and posterior tibial arteries' pulses were palpable and there was no motor or sensory deficit. Regarding his medical history, he suffered hypertension and peripheral venous insufficiency.

**Figure 2:**
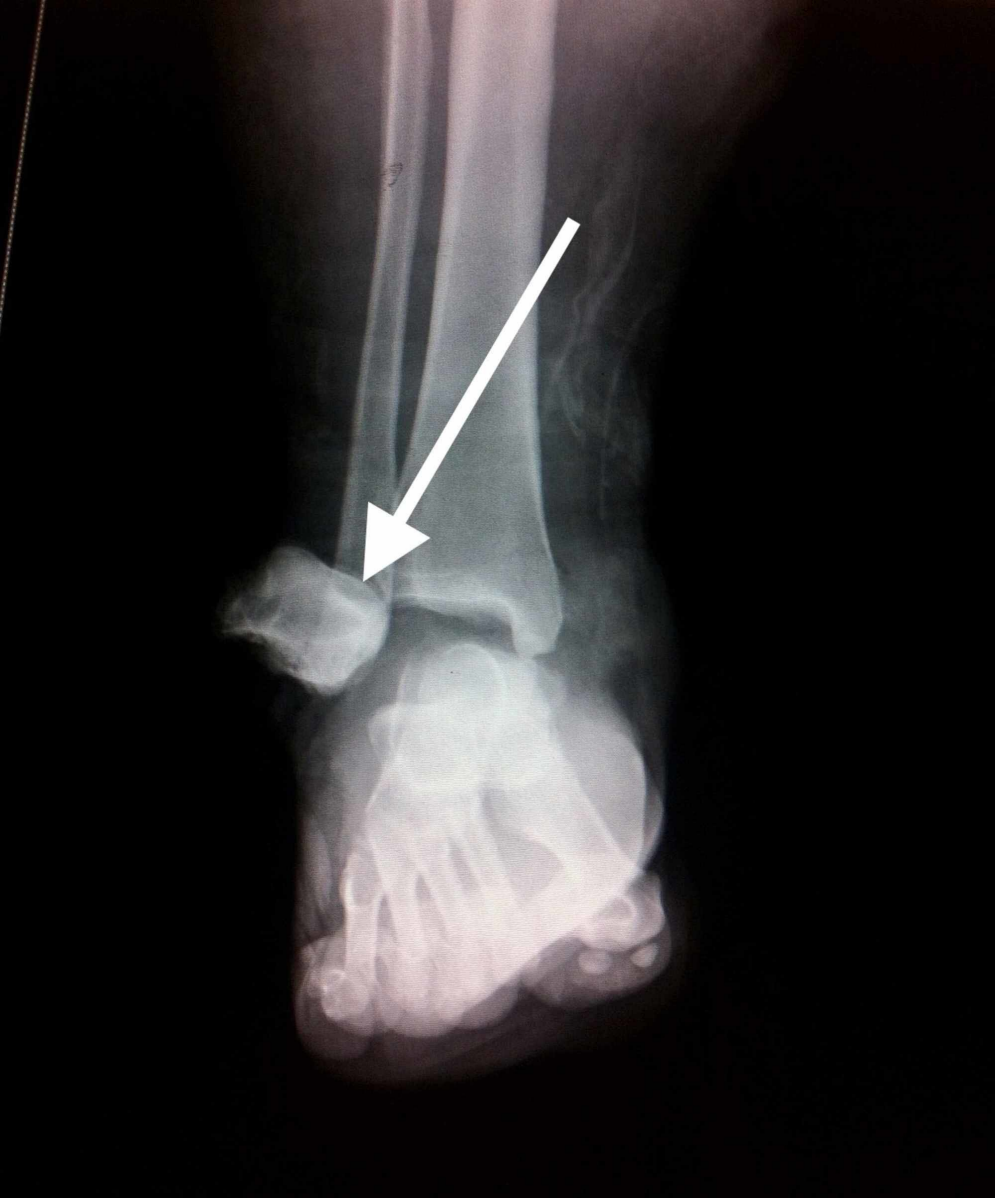
Preoperative antero-posterior radiograph of the right ankle demonstrating pan-talar dislocation (arrow)

**Figure 3:**
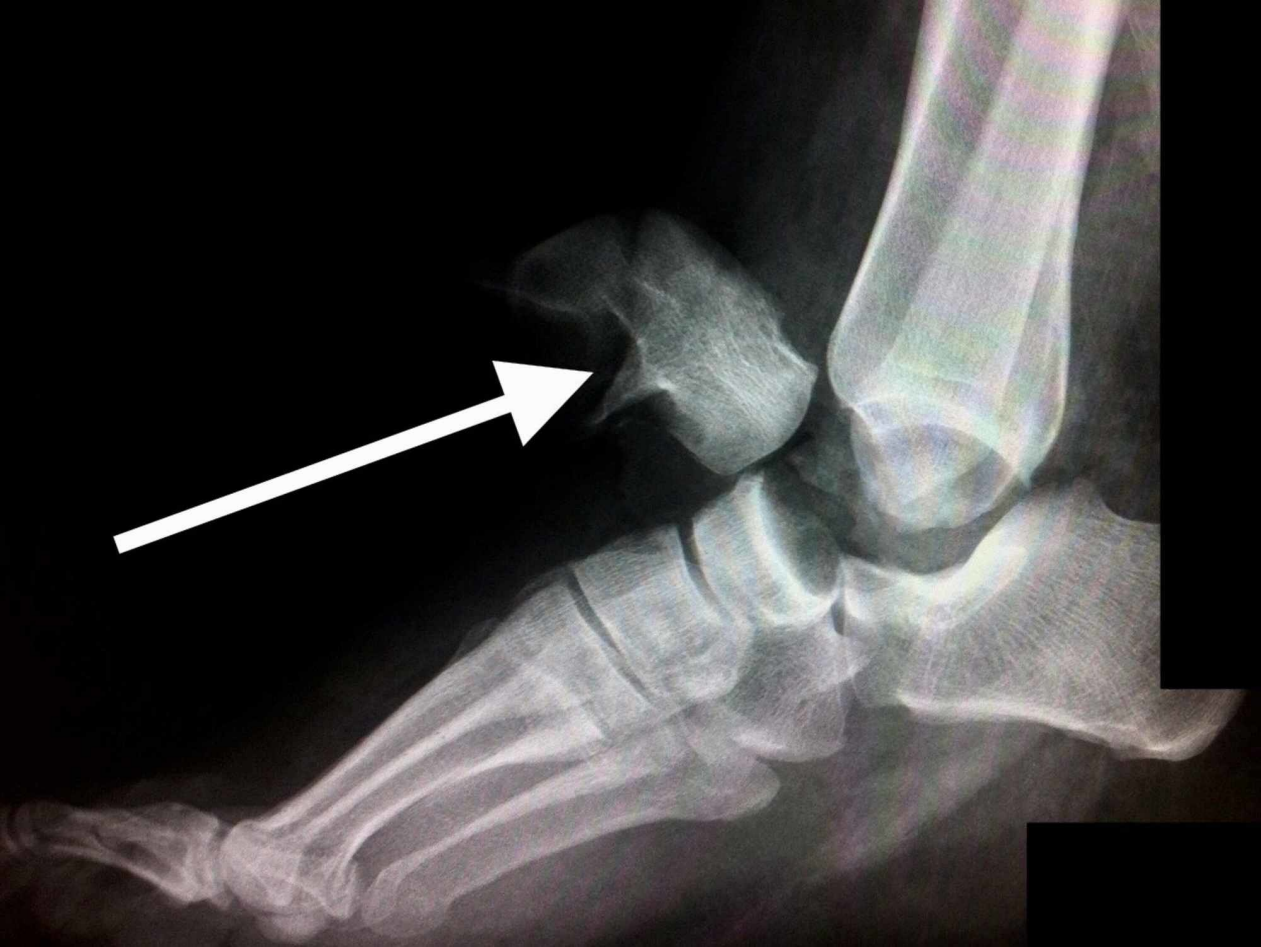
Preoperative lateral radiograph of the right ankle demonstrating pan-talar dislocation (arrow)

Plain radiographs were performed demonstrating total anterolateral talar dislocation (Figures 2, 3).

**Figure 4:**
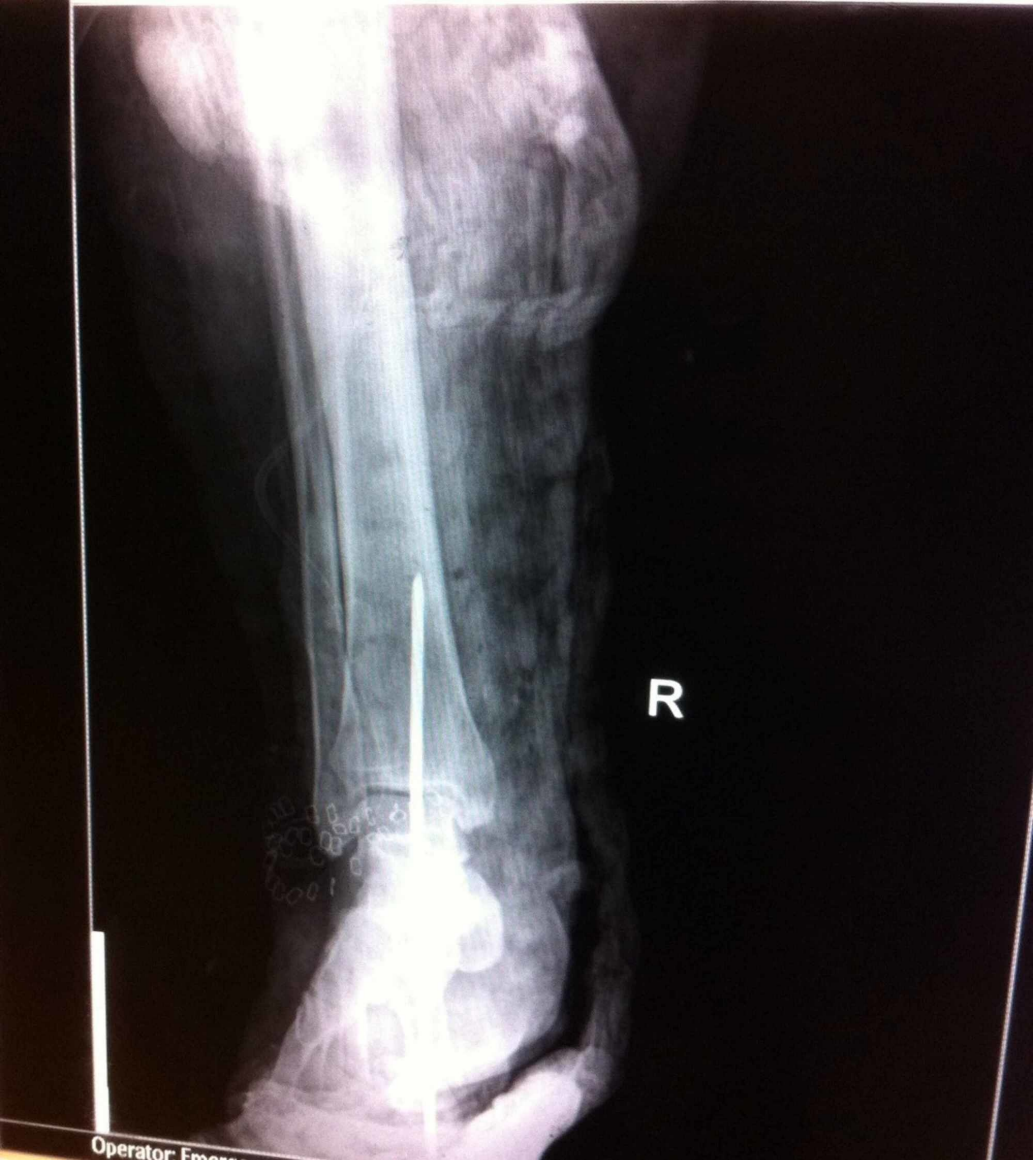
Postoperative antero-posterior radiograph of the right ankle demonstrating proper reduction of the talar dislocation and stabilization by the use of a Steinmann pin

In the emergency room, the wound was irrigated with one litre of normal saline. Tetanus toxoid booster and antibiotics were provided and the wound was dressed using an Aquacel® dressing. Within an hour, the patient was transferred to the operating theatre. Under general anaesthesia, the talus and the wound were irrigated with 9 litres normal saline. The dislocated talus was reduced in its anatomic position and held in place with one Steinmann pin placed from the inferior aspect of the calcaneus, through the talus and into the inferior aspect of the tibia under fluoroscopic guidance (Figure 4). After thorough wound debridement, and sterile saline-soaked gauze was applied. After 48 hours, second-look surgery for debridement and irrigation was done and wound coverage was successful using a skin graft.

**Figure 5:**
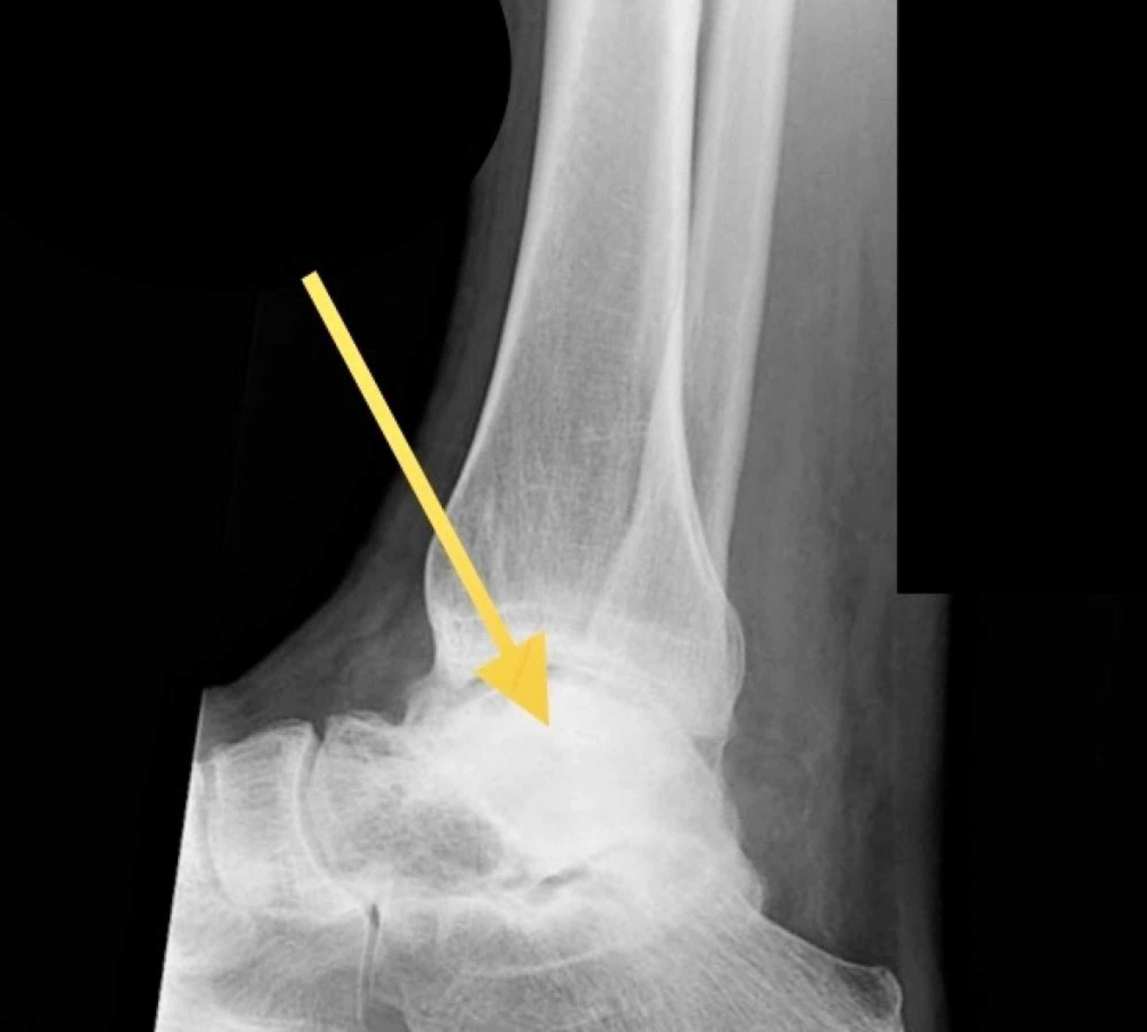
A lateral radiograph of the right ankle demonstrating signs of avascular necrosis of the talus (arrow)

Postoperatively, the patient was kept non-weight-bearing for six weeks in a back slab. Clips were removed after three weeks. At six weeks postoperative, the Steinmann pin was removed, and the patient was allowed to partial weight bear. Afterwards, he didn’t attend any of his follow-up appointments. He presented 12 months post-injury in December 2019 with a complaint of residual ankle pain which is controlled by daily medication. He claimed that he was able to gradually resume his level of daily activities at 14 weeks post-injury. Radiographs demonstrated signs of talar avascular necrosis (Figure 5). The patient’s ankle joint range of motion was 25 degrees of plantar flexion and 5 degrees of dorsiflexion. The patient could walk without aids and could squat, corresponding to an American Orthopaedic Foot and Ankle Society score of 75, involving a score of 30 points for pain, 35 for function, and 10 for alignment. The patient was counselled regarding treatment options in terms of ankle fusion to alleviate pain and that would affect his already good range of motion, although he still considering his options. Given that his outcome, the patient was kept on regular follow-up.

## Discussion

Pan-talar extrusion is a rare injury that results mostly from high-energy trauma [1-3]. Regarding the anatomy, 60% of the talus is covered with articular cartilage with no muscular attachments which make the talus vulnerable to dislocation. Extreme supination and plantarflexion forces result in talar dislocation out of the ankle mortise, with disruption of the strong ligamentous attachments, which can result in an open injury [4]. Most of the literature describes talar extrusion with an anterolateral wound, which is similar to our case report [5-9].

When facing a case of open talar extrusion, we should consider the possible complications including avascular necrosis, infection, post-traumatic arthritis and the need for secondary procedures [3-8]. Hiraizumi et al. concluded that the risk of avascular necrosis was the highest in the cases where no further attached soft tissue to the talus [9]. Development of avascular necrosis is very difficult to predict in the early postoperative phase. Hawkins’ sign is the only early predictive indicator of revascularization that can be seen on conventional radiography, where subchondral radiolucency in the talar dome is visible six to eight weeks post-injury [10].

Open talar extrusion is commonly complicated by soft tissue infection with a potential rate of infection ranges from 25% to 38% of cases [7,11] versus 88.9% initially reported by Detenbeck and Kelly [12]. However, infection risk has been minimized in the recent literature due to improvement in the wound care staged procedures and appropriate antibiotic therapy [6,8].

Traditionally, the literature described talectomy with tibio-calcaneal arthrodesis as the treatment of choice for an open total talar dislocation. However, recently talar reimplantation after thorough debridement demonstrated satisfying short and long-term clinical outcomes with the advantage of retaining the talar height and bone stock [3-8,11,12].

## Conclusions

Immediate reimplantation of an open talar dislocation is an established salvage procedure. A minimally invasive Steinmann pin fixation is a cheap alternative to fancy fixators and can preserve the already endangered soft tissue around the ankle. A close follow up is mandatory to pick up both early and late complication.
